# Two-Level Incremental Checkpoint Recovery Scheme for Reducing System Total Overheads

**DOI:** 10.1371/journal.pone.0104591

**Published:** 2014-08-11

**Authors:** Huixian Li, Liaojun Pang, Zhangquan Wang

**Affiliations:** 1 School of Computer Science and Engineering, Northwestern Polytechnical University, Xi’an, China; 2 Department of Computer Science, Wayne State University, Detroit, Michigan, United States of America; 3 School of Life Science and Technology, Xidian University, Xi’an, China; UMIT, Austria

## Abstract

Long-running applications are often subject to failures. Once failures occur, it will lead to unacceptable system overheads. The checkpoint technology is used to reduce the losses in the event of a failure. For the two-level checkpoint recovery scheme used in the long-running tasks, it is unavoidable for the system to periodically transfer huge memory context to a remote stable storage. Therefore, the overheads of setting checkpoints and the re-computing time become a critical issue which directly impacts the system total overheads. Motivated by these concerns, this paper presents a new model by introducing *i*-checkpoints into the existing two-level checkpoint recovery scheme to deal with the more probable failures with the smaller cost and the faster speed. The proposed scheme is independent of the specific failure distribution type and can be applied to different failure distribution types. We respectively make analyses between the two-level incremental and two-level checkpoint recovery schemes with the Weibull distribution and exponential distribution, both of which fit with the actual failure distribution best. The comparison results show that the total overheads of setting checkpoints, the total re-computing time and the system total overheads in the two-level incremental checkpoint recovery scheme are all significantly smaller than those in the two-level checkpoint recovery scheme. At last, limitations of our study are discussed, and at the same time, open questions and possible future work are given.

## Introduction

For large scale and long-running applications, system failures are inevitable. In the absence of any protective measures, the applications must be restarted from the beginning whenever the failures occur. This will lead to a large waste of system overheads and system resources. Therefore, the system fault-tolerant schemes are proposed to solve this problem [1], and one of them is the checkpoint recovery technology [2], [3], [4] which is a widely used and resultful fault-tolerant measure. During the running process of the task, the system saves the task execution states to a reliable storage device periodically. Therefore, it can recover itself from the last stored state whenever a failure occurs. This avoids the task restarting from the beginning, improves the system reliability greatly, reduces the system overheads significantly and shortens the task completion time.

In the checkpoint technology, the checkpoint placement frequency is important. If the checkpoint interval is too small, the overheads created by setting checkpoints will result in large system overheads. Conversely, if the checkpoint interval is too large, the re-computing time and recovery time will be too long in the event of a failure. In this case, the checkpoint recovery scheme cannot achieve the desired effects and reduce the system total overheads as expected. So, there is a tradeoff between the checkpoint placement frequency and the system total overheads. The traditional one-level checkpoint recovery scheme [5], [6], [7] involves only one type of checkpoint, where each checkpoint is designed to tolerate the worst failure scenario. Therefore, the overheads of one-level checkpoints are very large. In order to reduce the overheads of setting checkpoints and the total system overheads, Vaidya [8] presented the two-level recovery scheme. In this scheme, two types of checkpoints, namely the *N*-checkpoint and local checkpoint, are used to deal with the less probable failures and the more probable failures, respectively. The experimental analyses show that the two-level checkpoint recovery scheme can achieve lower system overheads than the one-level one.

When the two-level checkpoint recovery scheme is used to the large scale and long-running tasks, the system needs to periodically transfer huge data about its running state to a remote reliable storage. So, the overheads of setting checkpoints and the re-computing time have become a critical issue, which directly impacts the total overheads. In order to further reduce the system total overheads, we propose a two-level incremental checkpoint recovery scheme based on the two-level checkpoint recovery technology. The proposed scheme sets three types of checkpoints, namely *N*-checkpoint, *m*-checkpoint and *i*-checkpoint. The *N*-checkpoint is used to deal with the less probable or infrequent failures, while the *m*-checkpoint and *i*-checkpoint are used to deal with the more probable or frequent failures. The main contributions of this paper are listed as follows: (1) we introduce the third type of lightweight checkpoint and propose a new two-level incremental checkpoint model; (2) For the two-level incremental checkpoint model, we give the global optimal checkpoint frequency function and the checkpoint placement algorithm, which is independent of the specific failure distribution type; (3) we give a method to determine the optimal two-level incremental checkpoint placement strategy; (4) we give the placement strategies and the related conclusions for the Weibull distribution and exponential distribution respectively, and then illustrate the fact that the placement algorithm is independent of the specific failure distribution type. Experiment results show that compared to the two-level checkpoint recovery scheme, the proposed scheme significantly reduces the transfers of the storing contents, the overheads of setting checkpoints and the re-computing time, and thereby reduces the system total overheads.

The rest of this paper is organized as follows. In section 2, the related work is discussed. In section 3, the proposed two-level incremental checkpoint recovery scheme is described in details. Section 4 takes the Weibull distribution and exponential distribution as examples to illustrate how to compute the checkpoint placement time instants. In section 5, the experimental analyses and performance analyses are presented. In section 6, limitations of our study, open questions and possible future work are discussed. Finally, Section 7 presents the conclusion.

## Related Work

As the system scale grows larger and lager, the system reliability problem becomes more and more important. Scientists have predicted that in future high-performance and large-scale computing tasks, the most three difficult and growing problems will be avoiding, coping with, and recovering from failures [9]. Due to the fact that the computing of the tasks become more and more complex and the execution time become longer and longer, the failure becomes more and more frequent. If there is no fault tolerance mechanism, the applications must be re-started from the beginning whenever failures occur, which will result in unacceptable performance overheads, especially for long-running applications.

The checkpoint recovery technology is used to tolerate the system failures, guarantee the system reliability, and ensure the successful completion of the long-running tasks [2], [3], [4]. The basic idea of the checkpoint recovery technology can be described as follows: during the running process of the task, the computation state is saved into the storage medium as a checkpoint file every once in a while; the file is read to restore to the last stored state whenever a system failure occurs, which avoids the task restarting from the beginning, reduces the system overheads and guarantees the successful completion of the tasks. Checkpoint placement strategy is a key issue in checkpoint technology, which determines the system overheads. If the checkpoint interval is too small, the overheads created by setting checkpoints will result in large system overheads. Conversely, if the checkpoint interval is too large, the re-computing time and recovery time will be too long in the event of a failure, which also results that the checkpoint recovery scheme cannot achieve the desired effects and reduce the system total overheads as expected. Many researchers have worked on the checkpoint placement problem and given a lot of excellent solutions.

In the traditional one-level checkpoint model, Young [5] presented an optimal checkpoint and rollback recovery model, and obtained the first approximation of the optimal checkpoint interval by which the total waste time was minimized. Based on the Young’s work, Daly [6] has proposed a more accurate cost function, which improved the first order approximation to a higher order approximation and further reduced the system overheads. The main contributions of Young [5] and Daly [6] lie in that they took the cost function of the whole execution period into account and established a novel derivation principle for the optimal checkpoint interval. Unfortunately, in their models, both of them assumed that random failures follow a Poisson process with a constant failure which cannot adequately represent the actual failure characteristics [10]. By deducing the checkpoint frequency function which optimizes the expected overhead, Ling *et al.* [7] presented an optimal one-level placement strategy. In this way, Ling *et al.* make the one-level checkpoint recovery scheme independent of the specific distribution and can be used for any failure distribution.

Due to the high overheads of traditional one-level checkpoint technology, Oliner *et al.* [11] presented a cooperative checkpointing technology that can reduce the system overheads and improve the system robustness. The cooperative checkpointing schedules the basic checkpoint placements following the traditional Young’s one-level checkpoint model. The difference from Young’s model lies in the technique that they use to further reduce the checkpoint cost, that is to say, based on the risk estimation of system failures, some scheduled checkpoints are adaptively skipped. Therefore, the performance of their cooperative checkpointing depends on the accurate failure prediction, which is challenging [12], [13]. Elnozahy *et al*. [14] and Naksinehaboon *et al.* [15] have proposed the incremental checkpoint model, which sets a series of incremental checkpoints between the traditional full checkpoints. The incremental checkpoint only save the states that must be used during the recovery process or the changed states instead of the whole application states, so this model can reduce the overheads of setting checkpoints, and then reduce the total system overheads. In addition, Paun *et al.* [16] reduced the overheads of the incremental checkpoint scheme by using the optimal checkpoint frequency function, which also achieved good results. Although it was considered that the scalability problem could be solved well by the incremental checkpointing, the incremental checkpointing methods are not always practical, because most of the implementations need some system-level support in hardware and the underlying operating system. Therefore, to avoid the above implementation concerns, Agarwal *et al*. [17] presented a purely software-based incremental checkpoint technique by using the secure hash function. Their scheme does not need system-level support, because the computation of the hash function can be executed in software.

The traditional one-level checkpoint recovery scheme can reduce the system overheads, but it involves only one type of checkpoint, and each checkpoint in the one-level checkpoint recovery scheme is designed to tolerate the worst failure scenario. Therefore, the overheads of one-level checkpoints are very large. In order to reduce the overheads of setting checkpoints and the system total overheads, Vaidya [8] presented the classic two-level recovery scheme, which sets two types of checkpoints, namely *N*-checkpoint and local checkpoint. The *N*-checkpoint and local checkpoint are saved in stable storage and local disk respectively for different failures, and the overhead of setting an *N*-checkpoint is much larger than the local checkpoint. In Vaidya’s scheme, the failure is divided into permanent failure and transient failure, and the permanent failure must be recovered from the *N*-checkpoint. Vaidya’s scheme uses *N*-checkpoint with high setting overheads to deal with the less probable or infrequent failures and uses local checkpoint with low setting overheads to deal with the more probable or frequent failures. This makes the common failure be processed faster, and then reduces the system total overheads compared with the one-level checkpoint scheme. In order to obtain the optimal performance, Vaidya determined the two-level checkpoint placement strategy for exponential failure distribution by numerical search. However, in reality, the exponential distribution fails to give a good overall fit to the failure data, and sometimes other distribution types can give a better fit [10], [17], such as the Weibull distribution. Hence, a general two-level checkpoint placement strategy is needed, which not only can be applied to the exponential failure distribution, but also can be applied to other distribution types [18]. This problem is still an unsolved open problem in this field. Later, multi-level checkpointing system was proposed [19], which can be considered as the general model of the two-level checkpoint recovery scheme. Multi-level checkpointing can potentially deal with the case, that different components have different performances, by assigning different costs to different types of checkpoints and allowing adaptive resiliency between different levels. Generally, lightweight checkpoints are used to deal with the more probable or frequent failures, while more expensive checkpoints are used to deal with the less probable or infrequent failures.

Since then, researchers paid more and more attentions to the checkpoint recovery scheme, and lots of excellent works have been done in these years [20]. Hilton *et al*. [21] studied the method how to achieve minimal recovery to reduce the recovery overheads. By using the similar idea, Refs. [1], [22], [23], [24] also provided complementary techniques to reduce the error probability, thus the probability of rollbacks was reduced. Li *et al*. [25] proposed a fast restart mechanism for checkpoint/recovery protocols in networked environments, which is a complementary technique to the multi-level checkpointing system. Cores *et al*. [26] and Akkary *et al*. [27] studied the scalability of the checkpoint recovery scheme, and proposed techniques to reduce the recovery overheads when the scalability of the application grows. To further reduce the overheads of the checkpoint recovery process, Cores *et al*. [28] carry out the study on how to reduce the size of the checkpoint files. Also, for large-scale distributed systems, Wei *et al*. [29] studied the use of process clones towards localizing recovery, and they proved that their protocol can result in localized recovery involving a single group when clones are employed. Recently, diskless checkpoint has been introduced as a solution to avoid the I/O bottleneck of disk-based checkpoint [30], [31]. However, although this method works well, the encoding time, the dedicated resources and the memory overhead imposed by diskless checkpoint are significant obstacles against its adoption. Checkpoint schemes implemented on practical application systems have also been researched. Rusu *et al*. [32] proposed two different failure recovery schemes, which are based on the coordinated checkpointing and the uncoordinated checkpointing, respectively. Then, the performance comparison of these two schemes is made in effectiveness and overheads, and it shows that the first method is better than the second one due to its lower failure rates and smaller overheads. Khunteta *et al*. [33] presented the review of the algorithms, which have been reported for checkpointing approaches in mobile ad hoc network. Also, Rodríguez *et al*. [34] focused on the performance evaluation and studied the factors that impact the checkpoint recovery scheme, and pointed out meaningful conclusions about the state-of-the-art and future research trends in the rollback-recovery field. Rehman *et al*. [35] thought that for the system reliability, both software and hardware abstraction layers of a system should be involved and contribute its particular advantages towards highly-reliable hardware/software system, and at the same time they proposed a novel compilation technique for reliability-aware software transformations and instruction-level vulnerability estimation method. Henkel *et al*. [36] introduces the most prominent reliability concerns from today’s points of view and roughly recapitulates the progress in the community so far, which is very instructional.

## Method

In order to facilitate the description of the proposed scheme, some notations used frequently in this paper are summarized in Table 1.

**Table 1:** Notation.

Notation	Meaning
*O_n_*	Overhead of setting an *N*-checkpoint
*O_m_*	Overhead of setting an *m*-checkpoint
*O_i_*	Overhead of setting an *i*-checkpoint
*R_n_*	Recovery cost of an *N*-checkpoint
*T*_re-compute1_	Re-computing time when a permanent failure occurs
*T*_re-compute2_	Re-computing time when a transient failure occurs
*R_m_*	Recovery cost of an *m*-checkpoint
*R_i_*	Recovery cost of an *i*-checkpoint
*s*()	Checkpoint frequency function
*p_n_*	The probability that a permanent failure occurs
*m*	Number of *m*-checkpoints between two neighboring *N*-checkpoints
*n*	Number of *i*-checkpoints between two neighboring *m*-checkpoints
*I*(*i*)	The checkpoint interval between *t_i_* and *t_i_*_+1_
*k*	The re-computing time coefficient
*T*	The time that a failure occurs
*f*()	Probability Density Function

### 1 Model of Two-level Checkpoint Incremental Checkpoint Scheme

**Figure 1:**
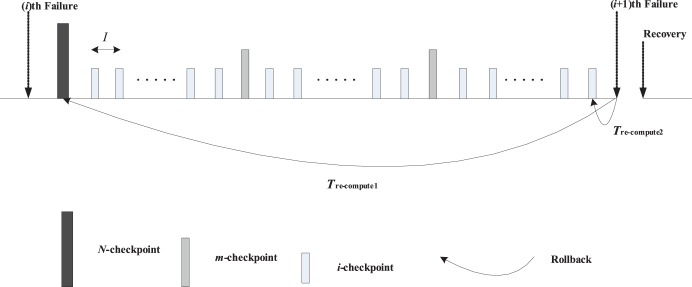
The two-level incremental checkpoint model.

The two-level incremental checkpoint model is shown in Fig. 1. The model contains three types of checkpoints, namely *N*-checkpoint, *m*-checkpoint and *i*-checkpoint. We describe the model in detail in the following.

In our model, the application sets *i*-checkpoints periodically, sets an *m*-checkpoint after *n i*-checkpoints periodically, and sets an *N*-checkpoint after *m m*-checkpoints periodically. The interval between two neighboring *N*-checkpoints is called a segment. The first checkpoint or the beginning checkpoint after a failure is always an *N*-checkpoint, which saves the total states in the remote stable storage. The remote stable storage is assumed to be always failure-free, so we can recover from the *N*-checkpoint no matter what type of failures occur. The *m*-checkpoint saves the application total states in the local disk. The overhead for saving application states in the local disk is much less than that in the remote stable storage. And, the recovery overhead from the local disk is also less than that from the remote stable storage. So when the transient failure occurs, we can recover from the *m*-checkpoint to reduce the system overheads. The *i*-checkpoint is also saved in the local disk, but it only saves the application states that have changed since the previous checkpoint. So the overheads of both setting *i*-checkpoint and recovering from *i*-checkpoint are quite low, which reduces the re-computing time significantly after the failure. We assume the overhead of setting an *N*-checkpoint, *m*-checkpoint and *i*-checkpoint is *O_n_*, *O_m_*, *O_i_*, respectively, and they meet *O_n_*>*O_m_*>>*O_i_*.

We divide the failures into permanent failure that occurs infrequently and transient failure that occurs frequently. The permanent failure is the one with low probability, and the transient failure is the one with high probability. When a permanent failure occurs, the application must recover from the *N*-checkpoint. Conversely, when a transient failure occurs, the application only need recover from the last *N*-checkpoint or *m*-checkpoint. If there are *i*-checkpoints after the last *N*-checkpoint or *m*-checkpoint, the application needs to read the *i*-checkpoint no matter from which checkpoint to recover. That is, when a failure occurs, the application can recover by the last *N*-checkpoint and several related *i*-checkpoints or the last *m*-checkpoint and several related *i*-checkpoints. The *i*-checkpoint can only be used with the *N*-checkpoint or the *m*-checkpoint in recovering the application and the sole *i*-checkpoint cannot recover any application. Thus, although there are three types of checkpoints, the two-level incremental checkpoint recovery scheme is not a three-level one as a particular case of [19]. In our paper, the overhead of recovering from *N*-checkpoint, *m*-checkpoint and *i*-checkpoint is *R_n_*, *R_m_*, *R_i_*, respectively, and they meet *R_n_*>*R_m_*>>*R_i_*.

Similar to [15], [16], [18], the following assumptions are also made in this paper.

1. The long-running application can be interrupted by a series of unexpected failures, and the failure follows the probability density function (PDF) *f*(*t*). And the failures are independent of each other.
2. The failure can be detected by a monitoring mechanism once the failure occurs.
3. The first checkpoint or the beginning checkpoint after a failure is always an *N*-checkpoint.
4. Because the process state is changing with the time, the size of the checkpoint file is constantly changing. In order to simplify the calculation, the overheads of setting checkpoints *O_n_*, *O_m_*, *O_i_* and the recovery cost of checkpoints are assumed to be constant. In practice, we use the average value of each parameter.
5. The number of *m*-checkpoint between two neighboring *N*-checkpoints is *m*, and the number of *i*-checkpoint between two neighboring *m*-checkpoints is *n*, and the *m* and *n* are both constant if no failures occur.
6. The failure never occurs during the re-computing and recovery time.

What we should point out is that although we adopt the similar assumptions used in [15], [16], [18], to simplify the problem, that is to say, assume that *m* and *n* are constant, they vary uncertainly and also affect the system performance. If *m* becomes larger, the overheads of setting checkpoints will become larger. If *m* becomes smaller, the overheads of re-computing time when a permanent failure occurs will become smaller. The value of *n* has the similar affect on system performance.

### 2 System Total overhead Function

The system total overhead *T*_total_overhead_ in the long-running application is consists of three parts [6], [16], [18]: the overhead of setting checkpoints *T*_set_checkpoint_, the re-computing time in the event of failures *T*_re-compute_ and the overhead of recovering from the failures *T*_recovery_. That is, *T*_total_overhead_ =  *T*_set_checkpoint_+*T*_re-compute_+*T*_recovery_. Next, we deduce the system total overhead function specifically in (0, *T*). We assume that the overheads corresponding to those three parts are *T*_set_checkpoint_(*T*), *T*_re-compute_ (*T*) and *T*_recovery_ (*T*) respectively in (0,*T*).

#### 2.1 Overhead of Setting Checkpoints

Due to the checkpoint placement procedure is a renewal process [37], therefore, the new cycle starts whenever a failure occurs. In order to obtain the optimal placement strategy of the two-level incremental checkpoint recovery scheme, we introduce the checkpoint frequency function. Here we first give the definition of the checkpoint frequency function, and then we deduce the overhead function of setting checkpoints of the two-level incremental checkpoint recovery scheme.

**Definition 1.** Let *s*(*t*) be checkpoint frequency function, then.
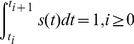
(1)where *t_i_*(*i* = 1,2,…) is the *i*th checkpoint placement, and *t*_0_ = 0.

We assume *T* is the time when a failure occurs. According to (1), the number of *N*-checkpoints, *m*-checkpoints and *i*-checkpoints in (0, *T*) are approximated by
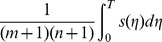
, 
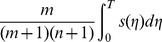
 and 
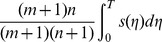
, respectively. So, in (0, *T*), the total overheads of setting checkpoints are.
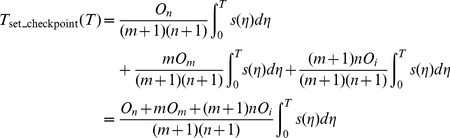
(2)


#### 2.2 The Re-computing time

The re-computing time is a period between the last recovery checkpoint and the present failure. For better dealing with the different scenarios, we divide the failures into two types. One is the permanent failure that is less probable and occurs infrequently, and the other is the transient failure that is more probable and occurs frequently. When a permanent failure occurs, the application must recover from the *N*-checkpoint. Conversely, when a transient failure occurs, the application can recover from the last *N*-checkpoint or *m*-checkpoint. If there are *i*-checkpoints after the last *N*-checkpoint or *m*-checkpoint, the application also needs to read the *i*-checkpoints no matter from which checkpoint to recover. We assume the probability of the permanent failure is *p_n_*. As shown in Fig. 1, when a permanent failure occurs, the re-computing time is *T*_re-compute1_, while when a transient failure occurs, the re-computing time is *T*_re-compute2_.

**Figure 2:**
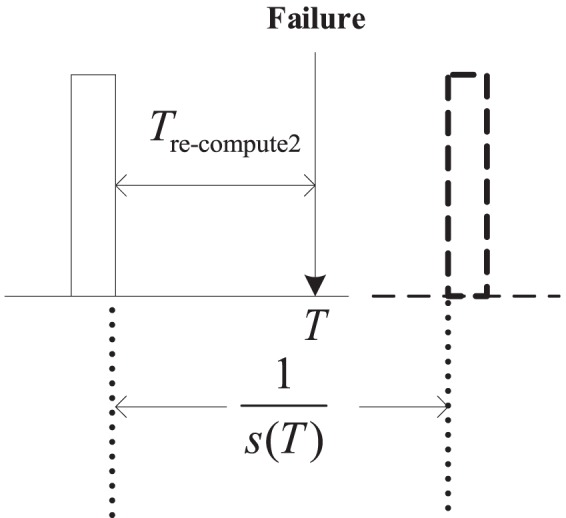
The relationship between *T*_re-compute2_ and the checkpoint interval.



When a transient failure occurs, the re-computing time *T*_re-compute2_ is the interval from last recovery checkpoint to the failure time. The relationship between *T*_re-compute2_ and the checkpoint interval is shown in Fig. 2. *T*_re-compute2_ can be expressed as (3) [18], where *k* is a re-computing time coefficient variable between (0, 1).

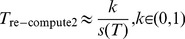
(3)


When a permanent failure occurs, the re-computing time *T*_re-compute1_ is the interval from last *N*-checkpoint to the failure time. *T*_re-compute1_ can be expressed as (4)
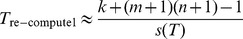
(4)


In summary, the total re-computing time can be expressed as.
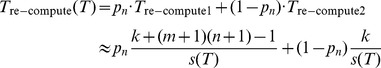
(5)


#### 2.3 Overhead of Recovering From Failures

The overhead of recovering from failures is the time consumed from reading the information from checkpoint to returning to the state that the last checkpoint saved after a failure occurs. According to assumption 4, the recovery cost of *N*-checkpoint, *m*-checkpoint and *i*-checkpoint, namely *R_n_*, *R_m_*, *R_i_*, are assumed to be constant. We assume the probability that a failure is permanent is *p_n_*, so the probability that a failure is transient is (1-*p*_n_). Then, the overhead of recovering from failures can be expressed as.
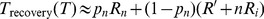
(6)where 
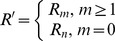
.

### 3 Optimal Checkpoint Frequency Function

Here, we first give the definition of the system total checkpoint overheads function, and then we deduce the global optimal checkpoint frequency function through the total checkpoint overheads function.

**Definition 2.** The total checkpoint overheads can be expressed as a function about the failure time *T*, which can be expressed as.
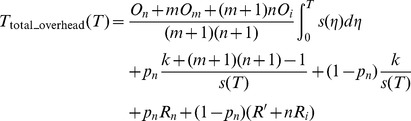
(7)where 
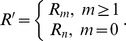


The time when a failure occurs is random during the application execution, so whenever a failure occurs, the application will recover from the corresponding checkpoint and place the new checkpoints, and the task will be also restarted after failures. Therefore, checkpoint placement process is a renewal reward process. We define *W_i_* as the total overheads from the starting or restarting point to the *i*th failure. The total overheads of the long-running application can be expressed as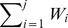, where *j* is the number of failures. According to the theorem of a renewal reward process [37], we obtain.
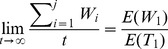
(8)


*T*_1_ is the time when the first failure occurs. The left hand side of the above equation represents the total average overheads, and it is a function of the average overheads in the first circle, *E*(*W*_1_). The Equation (8) suggests that minimizing the total average overheads is equivalent to minimizing the overheads from the starting point to the first failure. We define *f*(*t*) as the probability density function of the failure, then the average checkpoint overhead in the first circle is described as follows:
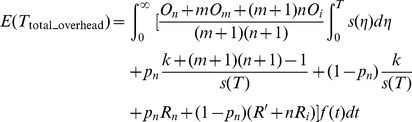
(9)


By solving the minimum of (9), we can get the optimal checkpoint frequency function *s*(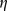)_opt_.

The conclusion of the optimal checkpoint frequency function is shown as Theorem 1.

**Theorem 1**. *The optimal checkpoint frequency function that minimizes the global average checkpoint overhead can be expressed as*

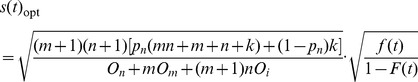
(10)


**Proof.** Let 
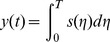
. By substituting it into (9), we obtain
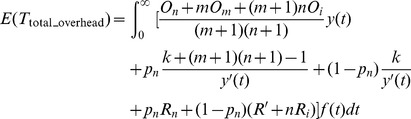
(11)


We let
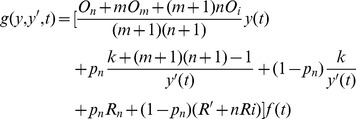
(12)


Based on the theorem of calculus of variations [15], if the integral in (12) has a minimum value, (12) must satisfy Euler-Lagrange in (13)
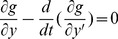
(13)


Taking the partial derivative of *g* with respect to *y* and *y*’ respectively, we have
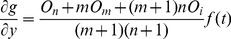
(14)

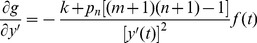
(15)


By substituting (14) and (15) into (13) and integrating on both sides of (15) on the interval (0, *t*), we obtain 
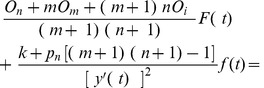
(16)where *C* is a constant. Because the function *y*(*t*) satisfies the conditions in the following.



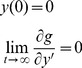
(17)


Applying the second condition in (17) to (16), we obtain.
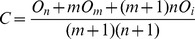
(18)


By substituting (18) into (16) we can get.
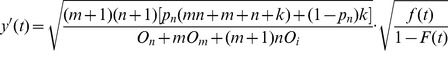
(19)


Because *s*(*t*) = *y*’(*t*), the optimal checkpoint frequency function that minimizes global average checkpoint overhead can be expressed as (10).

After obtaining the global optimal checkpoint frequency function, the checkpoint number *m*, *n* and the average checkpoint overhead *E*(*T*_total_overhead_) of the two-level incremental checkpoint placement strategy are determined. We can compute the optimal checkpoint placement time through the optimal checkpoint frequency function. If *k* and the minimum of *m*, *n* are obtained, the checkpoint placement strategy is determined finally. Before determining the checkpoint placement time we should give the method to estimate the expected re-computing time coefficient *k*.

### 4 Estimation of Expected Re-computing Time Coefficient
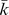


As shown in Fig. 2, we can use the re-computing time *T*_re-compute2_ and the checkpoint interval to estimate the re-computing time coefficient *k*. In addition, it is obvious that the re-computing time *T*_re-compute2_ is a random variable depending on the time when the failure occurs. Therefore, if we know the distribution of the time between failures, then *T*_re-compute2_ can be estimated, and then *k* also can be estimated.

**Definition 3.** The re-computing time coefficient *k* is the ratio between the re-computing time *T*_re-compute2_ and the checkpoint interval in which a failure occurs. So, the re-computing time coefficient *k* can be expressed as.
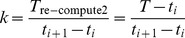
(20)where *T* is the time when the failure occurs. In order to estimate *k*, we first need the following definition to estimate the expected re-computing time *T*_re-compute2_ for each checkpoint interval.

**Definition 4.** Excess life is a random variable, *Z*>0, which denotes system survival until time *t*+*Z* given that it survives till time *t*. We respectively denote the cumulative distribution function (CDF), the probability density function (PDF) and the expected value of the excess life *Z* as follows.
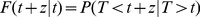
(21)

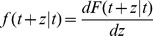
(22)

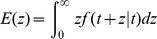
(23)


In our model, *t_i_* is the *i*th checkpoint placement. The re-computing time *T*_re-compute2_ during the interval is a random variable such that its value is in the interval (0,*t_i+_*_1_-*t_i_*). According to Definition 4, the expected value of re-computing time *T*_re-compute2_ can be expressed as.
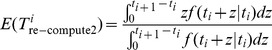
(24)


Therefore, the expected *k* of the *i*th checkpoint interval, 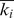, is.
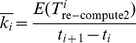
(25)


Hence, the expected re-computing time coefficient is.
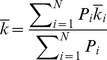
(26)where *P_i_* = *P*(*t_i_*<*T*<*t_i+_*_1_ *|T*>*t_i_*) and *N* is the number of the checkpoints. The method to estimate the re-computing time coefficient *k* is given by (26), therefore the minimum of *m* and *n* is obtained, and then the two-level incremental checkpoint placement strategy is determined finally. Next, we give a method to determine the two-level incremental checkpoint placement strategy.

### 5 Determine Two-level Incremental Checkpoint Placement Strategy

From (10) we can see that the re-computing time coefficient *k*, the number of *m*-checkpoint *m* and the number of *i*-checkpoint *n* are closely related, and therefore, in practice, we have to find *k*, *m* and *n* at the same time. In the following we give the method to estimate *k* and the minimum of *m* and *n*.

**Algorithm 1.** Algorithm to estimate *k* and the minimum of *m*, *n*:

Step 1: Initialize the parameter *k*, *m* and *n*. Let *k*_ini_ = 0.5, *m*_ini_ = 1, *n*_ini_ = 1. (when *m* = 0 or *n* = 0 the two-level incremental checkpoint recovery scheme degenerates to the two-level checkpoint recovery scheme, so the value *m* = 0 or *n* = 0 has no meanings.).

Step 2: Input *k*_ini_, *m*_ini_ and *n*_ini_. Calculate the optimal checkpoint frequency function using (10). Output *s*(*t*)_opt_.

Step 3: Input *s*(*t*)_opt_. Calculate the minimum of *m* and *n* using (9). Output *m*_min_ and *n*_min_.

Step 4: Input *k*_ini_, *m*_min_ and *n*_min_. Calculate the checkpoint placement time relating to *k*_ini_, *m*_min_ and *n*_min_ using (1) and (10). Output *t*_1_, *t*_2_,…, *t_N_*.

Step 5: Input *t*_1_, *t*_2_,…, *t_N_*. Calculate the expected re-computing time coefficient using the (24), (25) and (26). Output 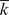.

Step 6: If 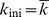, set 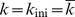, the algorithm ends. Otherwise, set 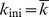, and return to step 2.

When *k* and the minimum of *m* and *n* are determined, the checkpoint placement time can be calculated using (1), and then the two-level incremental checkpoint placement strategy is determined finally.

We can see that the above derivation processes about the optimal frequency function *s*(*t*)_opt_, *k* and the minimum of *m* and *n* are not specific for a certain kind of failure distribution, but only involve the abstract form of distribution functions *f*(*t*). Therefore, the checkpoint placement method does not depend on specific failure distribution types, and the method can be applied to different failure distribution types, such as the Weibull distribution, exponential distribution and so on. When the method is applied to specific failure distribution type, we only need to replace *f*(*t*) with the specific failure distribution.

## Examples

Because the two-level incremental checkpoint recovery scheme proposed in this paper is independent of the failure distribution type, it is applicable to different failure distribution types. And thus, we can calculate the checkpoint placement time under any failure distribution type. Although the failure distribution types are various, the methods to calculate the checkpoint placement time for different distribution are similar. The Weibull distribution and exponential distribution fit the actual failure features best, and therefore, we take them as examples to illustrate how to calculate the two-level incremental checkpoint placement time, and then to determine the checkpoint placement strategy.

Whether the failure follows the Weibull distribution or exponential distribution, when we want to determine the checkpoint placement time using the algorithm mentioned in Section 3.4, we first need to calculate the minimum of *m* and *n*, namely *m*_min_ and *n*_min_, respectively. About calculating *t*_1_, *t*_2_,…, *t_N_* in step 4, we give the following conclusions.

In order to calculate the checkpoint placement time better, we first give CDF and PDF of the Weibull and exponential distribution. The CDF and PDF of the Weibull distribution are 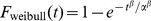
and 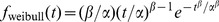, respectively, where 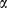 is the scale parameter and 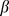 is the shape parameter. The CDF and PDF of the exponential distribution are 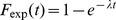
and 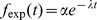, respectively, where 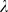 is the rate parameter.

**Theorem 2.** *Let t_i_(i = 1,2,…) be the checkpoint placement time, such that t_0_ = 0. When the failure distribution follows the Weibull distribution, t_i_ can be expressed as*

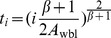
(27)
*where* 
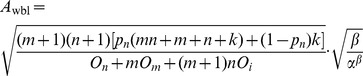
.

**Proof.** When the failure distribution follows the Weibull distribution, by substituting the CDF and PDF of the Weibull distribution into (10), we obtain the optimal checkpoint frequency function for the Weibull distribution.
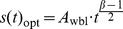
(28)where 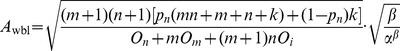. According to Definition 1, we have.
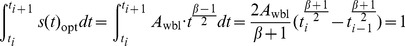
(29)


Therefore,
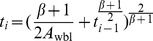
(30)


By induction, we obtain.
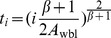
(31)where *i* = 0,1,2,…, and *t*_0_ = 0.

**Theorem 3.** *Let t_i_(i = 1,2,…) be the checkpoint placement time, such that t_0_ = 0. When the failure distribution follows the exponential distribution, t_i_ can be expressed as*

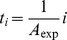
(32)
*where* 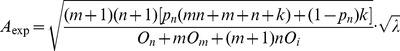.

**Proof.** When the failure distribution follows the exponential distribution, by substituting the CDF and PDF of the exponential distribution into (10), we obtain the optimal checkpoint frequency function for exponential distribution.
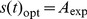
(33)where 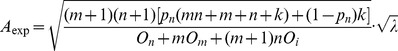.

According to Definition 1, we have
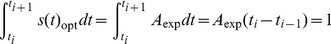
(34)


Therefore,
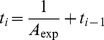
(35)


By induction, we obtain.
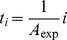
(36)where *i* = 0,1,2,…, and *t*_0_ = 0. 

Using Theorem 2 and Theorem 3, we can calculate the checkpoint placement time for the Weibull distribution and exponential distribution. Next, we analyze the changes between two neighboring checkpoint intervals when the failure follows the Weibull distribution and exponential distribution respectively.

**Theorem 4.** *Let I(i) be the checkpoint interval between t_i_ and t_i+1_. When the failure follows the Weibull distribution, if the shape parameter 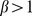, I(i) is decreasing, and if the shape parameter 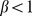, I(i) is increasing.*

**Proof.** According to (27), when the failure follows the Weibull distribution, we have
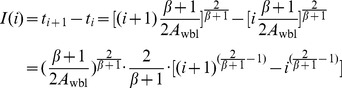
(37)where 
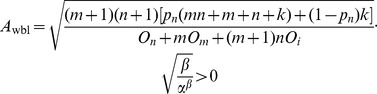
.

Solving the first derivation of *I*(*i*), we have
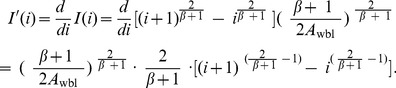
(38)


From (38), we can see that if 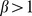, *I’*(*i*)<0, that is to say, the checkpoint interval *I*(*i*) is decreasing, and if 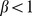, the checkpoint interval *I*(*i*) is increasing.

Note: If 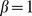, the Weibull distribution turns into exponential distribution, the related conclusions are shown in the following.

**Theorem 5.** *Let I(i) be the checkpoint interval between t_i_ and t_i+1_. When the failure follows the exponential distribution, I(i) is constant unrelated with the i.*

**Proof.** According to (27) when the failure follows the exponential distribution, we have.
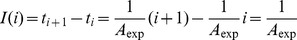
(39)where 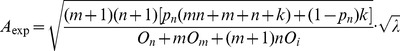.

From (38), we can see that *I*(*i*) is constant unrelated with the *i*.

Here, we only take the Weibull distribution and exponential distribution as examples to illustrate how to calculate the two-level incremental checkpoint placement time, and analyze the nature of the checkpoint interval for the Weibull distribution and exponential distribution. When the failure follows other distribution types, the checkpoint placement time also can be calculated by (1).

After the checkpoint placement time for the Weibull distribution and exponential distribution are calculated by (27) and (32), the expected re-computing time coefficient 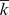 can be calculated using the step 5 of the algorithm in Section 3.4, and then through the judgment of step 6, the *k*, *m*_min_, *n*_min_ and the checkpoint placement sequences *t*_1_, *t*_2_,…, *t_N_* can be determined. In this way the checkpoint placement strategy for the Weibull and exponential distribution is determined finally.

## Performance Analyses

For the two-level checkpoint recovery scheme which is used to the large scale and long-running tasks, huge memory context must be transferred through the network and saved in a reliable storage. So the overheads of setting checkpoints and the re-computing time directly impact the system total overheads and the system performance. In order to further reduce the overheads of setting checkpoints, the re-computing time, the system total overheads, and make the scheme be applied to any type of failure distribution, we present a two-level incremental checkpoint recovery scheme based on the ideal that using checkpoint with high setting overheads to deal with the less probable or infrequent failures and using checkpoint with low setting overheads to deal with the more probable or frequent failures.

However, for the traditional one-level checkpoint recovery scheme, the two-level checkpoint recovery scheme and our two-level incremental checkpoint recovery scheme, they all have their own merits or demerits. In the one-level checkpoint recovery scheme, either full or incremental checkpoint, only one kind of failure has been taken into account, and thus its placement strategy is simple. When a failure occurs, what we should do is just to recover the system for the latest checkpoint. However, this method cannot deal with different failures in different way, which forecloses the aim of the optimal performance. Compared with the one-level checkpoint recovery scheme, the two-level scheme can deal with the two different failures and achieve the more optimal performance. But it makes the placement strategy more difficult, because two kinds of checkpoints should be considered. Besides, it cannot distinguish the failures with different frequency. Our two-level incremental checkpoint recovery scheme adopted three kinds of checkpoints to deal with the above failures to achieve the optimal performance. But, the larger the number of kinds of checkpoints, the more difficult the checkpoint placement strategy becomes. In a word, compared to the two-level checkpoint recovery scheme, the proposed scheme significantly reduces the overheads of setting checkpoints and the re-computing time, and thereby reduces the system total overheads. In addition, this paper deduces the global optimal checkpoint overheads function and solves the problem that how to determine the optimal checkpoint placement strategy through.

To evaluate performance of our scheme, in this section, we first discuss the factors affecting the number of *i*-checkpoints between two neighboring *N*-checkpoints, and then analyze and show the advantages of the two-level incremental checkpoint recovery scheme compared with the two-level checkpoint recovery scheme.

### 1 Factors Affecting Optimal Number of Checkpoint in One Segment

The number of *i*-checkpoints between two neighboring *N*-checkpoints is the key factor that determines the checkpoint placement and affects the re-computing time and the system total overheads. If the number of *i*-checkpoints is obtained, with the value of the parameter *p_n_*, we can obtain the related number of *m*-checkpoints. In this case, the checkpoint placement strategy is determinate. So, we will mainly give a mathematical analysis and conclusions about the optimal number of *i*-checkpoints between two neighboring *N*-checkpoints in our checkpoint placement strategy.

**Figure 3:**
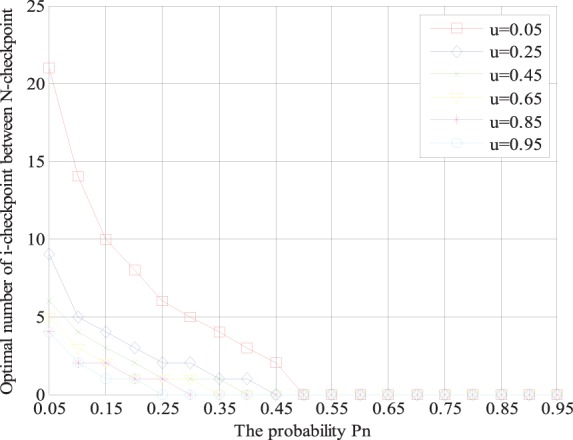
The relationship between optimal number of *i*-checkpoints and *p*_n_ under different *u* = *O_i_/O_m_*.

According to Section 3.3, we can determine the optimal number of *i*-checkpoints between two neighboring *N*-checkpoints with specific parameters. The tendency of the optimal number of *i*-checkpoints will be shown visually by several groups of examples in Fig. 3. The parameter *p_n_* is the probability of recovering from an *N*-checkpoint, and *u = O_i_/O_m_* is the ratio of the overheads of setting *i*-checkpoint and *m*-checkpoint. The range of *u* is (0,1). Note that we do not care the occasions when *u* = 0 and *u* = 1. When *u* = 0, it means the overhead of *i*-checkpoint is 0; when *u* = 1, it means the overhead of *i*-checkpoint is equivalent to the overhead of *m*-checkpoint. In both cases, the two-level incremental checkpoint recovery scheme will degenerate to the two-level checkpoint recovery scheme. The range of *p_n_* is (0, 1). The case, *p_n_* = 0 or *p_n_* = 1, means only permanent failure or only transient failure occurs in the system, which does not match the actual situations, so we do not consider these two cases either. In fact, during the practical running, the checkpoint recovery scheme must be affected by other factors, such as network throughout and I/O interaction [30], [31]. However, the existing schemes [15], [16], [18] just considered the main factors that affect the system performance basically and their experiments ignore the affection of them. Now, there have been some other researches that study their affection on the checkpoint recovery scheme, which has been considered as another new and independent research topic. Also in our experiment, to achieve the performance comparison between the existing schemes and ours in the same circumstance, we also ignore these factors like [15], [16], [18]. Studies of the affection of these factors is not our contribution of this paper, and may be one of our further works.

Fig. 3 shows that the optimal number of *i*-checkpoints between two neighboring *N*-checkpoints varies with the parameter *p_n_* for a given value *u* according to our placement strategy. As shown in Fig. 3, for a given value *u*, the greater probability *p_n_* is, the smaller the optimal number of *i*-checkpoints is. When *p_n_* is large enough, no *i*-checkpoint is taken. For example when *u* = *O_i_/O*_m_ = 0.05 and *p_n_* = 0.5, there is no *i*-checkpoint in the proposed scheme and there only exist *N*-checkpoint and *m*-checkpoint, and now the two-level incremental checkpoint recovery scheme degenerates to the two-level checkpoint recovery scheme. This is because when the probability *p_n_* of the permanent failure rises, which means that the permanent failure occurs frequently. And in this case, the system only can recover from the *N*-checkpoint, which results in that the placement of *i*-checkpoint becomes less and less. In order to reduce the system overheads, the *i*-checkpoint should be set less and less until it disappears.

**Figure 4:**
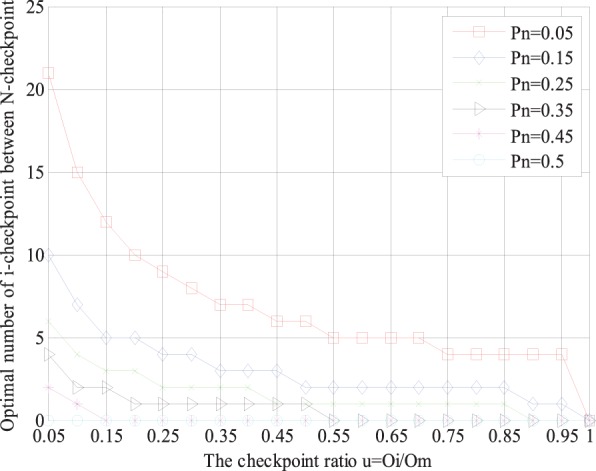
The relationship between optimal number of *i*-checkpoints and *u* under different *p_n_*.

The following Fig. 4 shows that the optimal number of *i*-checkpoints between two neighboring *N*-checkpoints varies with the checkpoint ratio *u* for a given value *p_n_* according to our placement strategy.

As shown in Fig. 4, the comparison results of several curves of different value *p_n_* show that that the greater checkpoint ratio *u* is, the smaller the optimal number of *i*-checkpoint for a given parameter *p_n_* is. Especially when the value *u* is large enough, no *i*-checkpoint is scheduled. For example, when *p_n_* = 0.25 and *u* = *O_i_/O_m_* = 0.9, there will be no *i*-checkpoints and there only exist the *N*-checkpoint and *m*-checkpoint, and now the two-level incremental checkpoint recovery scheme degenerates to the two-level checkpoint recovery scheme. This is because when the value *u* becomes larger and larger, which results that the overheads of setting *i*-checkpoints become larger and larger and gradually approach the overheads of setting *m*-checkpoint. The above situation results in that the contents of *i*-checkpoint are approximately equal to those of *m*-checkpoint, so the *i*-checkpoint gradually changes to *m*-checkpoint until it disappears.

### 2 Performance Comparisons

In this section, we use three groups of experiments to analyze the advantages of two-level incremental checkpoint recovery scheme compared to the two-level checkpoint recovery scheme. All these three groups of experiments are carried out under the Weibull distribution and exponential distribution. The first group of experiments compares the total overheads of setting checkpoints, the total re-computing time, the total overheads of recovering from failures, the system total overheads with the numbers of the failure between the two-level increment checkpoint recovery scheme and the classical two-level checkpoint recovery scheme [8]. The second group of experiments compares the total overheads of setting checkpoints, the total re-computing time, the total overheads of recovering from failures, the system total overheads with the task completion time between these two schemes. The third group of experiments compares the total overheads of setting checkpoints, the total re-computing time, the total overheads of recovering from failures, the system total overheads with the numbers of the failure under the different checkpoint ratio *u* between these two schemes. The system total overheads refer to the sum of total overheads of setting checkpoints, total re-computing time and total overheads of recovering from failures.

Our simulations are based on the 22 high-performance computing systems in LANL (Los Alamos National Labs) from February 23, 1997 to September 2, 2004, which is a period of round 3,958,008 minutes and has 514 failures. When the failure follows the exponential distribution, like [8], we also assume the rate parameter of permanent failure *λ_p_* = 10^−5^ and the rate parameter of transient failure *λ_l_* = 10^−6^. When the failure follows the Weibull distribution, we make the best fitted Weibull distribution to the Node1’s failure datum of System2 in LANL from February 23, 1997 to December 10, 2004, and obtain the fitted shape parameter 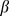 = 0.6857 and the scale parameter 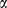 = 2.0815. And the other parameters are shown in Table 2.

**Table 2:** The parameters of two-level incremental checkpoint recovery scheme.

Parameter	*p_n_*	*k*_ini_	*O_n_*	*O_m_*	*O_i_*	*R_n_*	*R_m_*	*R_i_*
Value	0.05	0.5	1.0	0.1	0.005	1.0	0.1	0.005

#### 2.1 Performance Comparisons under the Failure Numbers

**Figure 5:**
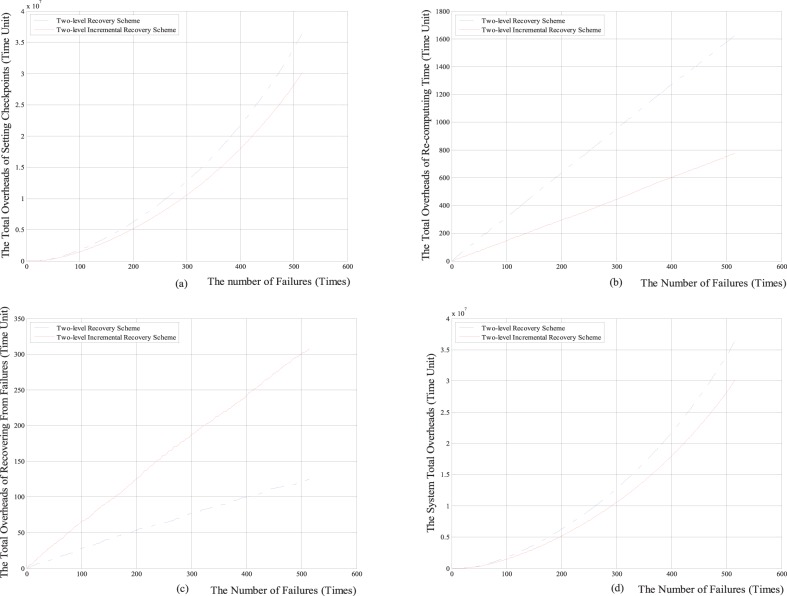
The comparison results between two-level incremental checkpoint recovery scheme and two-level checkpoint recovery scheme for the Weibull distribution. (a) The relationship between the total overheads of setting checkpoints and the number of failures; (b) The relationship between the total re-computing time and the number of failures; (c) The relationship between the total overheads of recovering from the failures and the number of failures; (d) The relationship between the system total overheads and the number of failures; Note: Time Unit depends on parameters in practical implementation, such as the practical value of *O_m_*, so it is not given here, which is similar to the case in Figs. 6–10.

**Figure 6:**
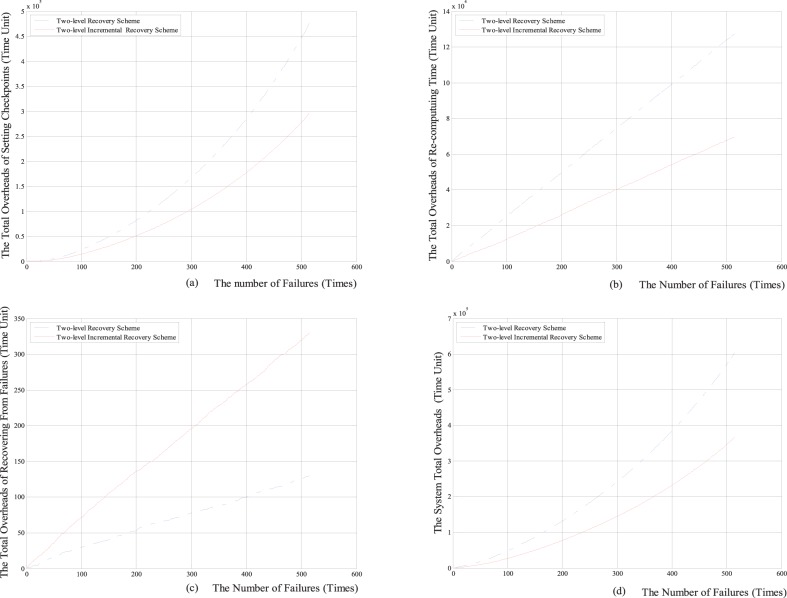
The comparison results between two-level incremental checkpoint recovery scheme and two-level checkpoint recovery scheme for exponential distribution. (a) The relationship between the total overheads of setting checkpoints and the number of failures; (b) The relationship between the total re-computing time and the number of failures; (c) The relationship between the total overheads of recovering from the failures and the number of failures; (d) The relationship between the system total overheads and the number of failures.

Firstly, we respectively compare the total overheads of setting checkpoints, the total re-computing time, the total overheads of recovering from failures and the system total overheads with the numbers of the failure between the two-level increment checkpoint recovery scheme and the classical two-level checkpoint recovery scheme. The comparison results are shown in Fig. 5 and Fig. 6.

From Fig. 5 and Fig. 6, we can see that for both the Weibull distribution and exponential distribution, the total overheads of setting checkpoints, the total re-computing time and the system total overheads in our two-level incremental checkpoint recovery scheme are all less than those in the two-level checkpoint recovery scheme. Only the total overheads of recovering from failures in our two-level incremental checkpoint recovery scheme is slightly larger than the two-level checkpoint recovery scheme, this is because when the transient failure occurs, the two-level checkpoint recovery scheme needs to read the *i*-checkpoint after the last *N*-checkpoint or *m*-checkpoint, which increases the recovery overheads. However, the growth of total overheads of recovering from failures is negligible compared to the reduction of the other aspects.

#### 2.2 Performance Comparisons under the task completion time

**Figure 7:**
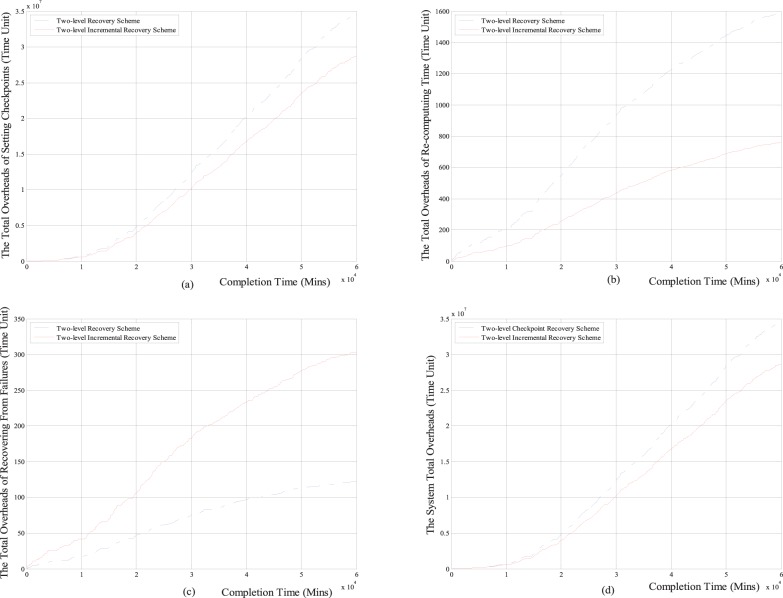
The comparison results between two-level incremental checkpoint recovery scheme and two-level checkpoint recovery scheme for the Weibull distribution. (a) The relationship between the total overheads of setting checkpoints and the completion time; (b) The relationship between the total re-computing time and the completion time; (c) The relationship between the total overheads of recovering from the failures and the completion time; (d) The relationship between the system total overheads and the completion time.

**Figure 8:**
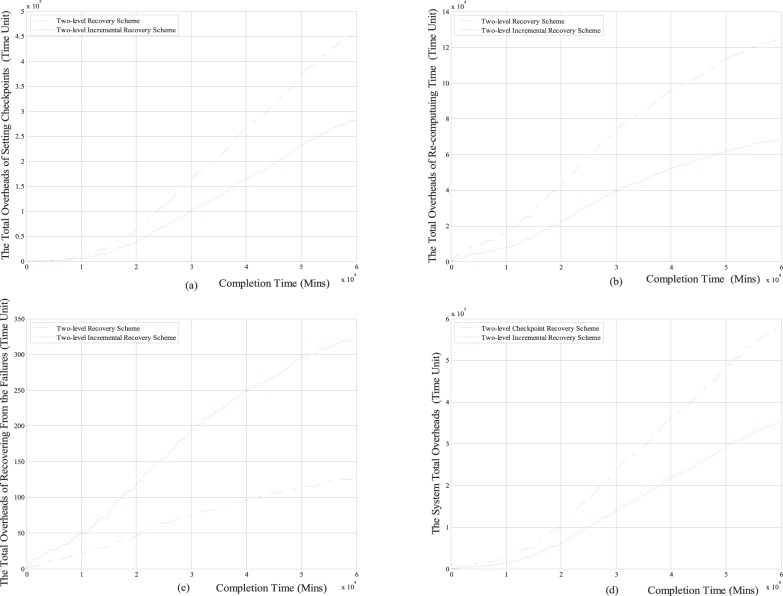
The comparison results between two-level incremental checkpoint recovery scheme and two-level checkpoint recovery scheme for exponential distribution. (a) The relationship between the total overheads of setting checkpoints and the completion time; (b) The relationship between the total re-computing time and the completion time; (c) The relationship between the total overheads of recovering from the failures and the completion time; (d) The relationship between the system total overheads and the completion time.

Next, we respectively compare the total overheads of setting checkpoints, the total re-computing time, the total overheads of recovering from failures and the system total overheads with the task completion time between the two-level increment checkpoint recovery scheme and the classical two-level checkpoint recovery scheme. The comparison results are shown in Fig. 7 and Fig. 8.

From Fig. 7 and Fig. 8, we can see that for both the Weibull distribution and exponential distribution, the total overheads of setting checkpoints, the total re-computing time and the system total overheads in our two-level incremental checkpoint recovery scheme are all less than the two-level checkpoint recovery scheme. Only the total overheads of recovering from failures in our two-level incremental checkpoint recovery scheme is slightly larger than the two-level checkpoint recovery scheme, this is also because when the transient failure occurs, the two-level checkpoint recovery scheme needs to read the *i*-checkpoint after the last *N*-checkpoint or *m*-checkpoint, which increases the recovery overheads. However, the growth of total overheads of recovering from failures is negligible compared to the reduction of the other aspects. And the longer the task completion time is, the larger the advantage of our proposed scheme in reducing the system total overheads is, which shows that our proposed recovery scheme is more suitable for long-running application task and can obtain the lower system total overheads.

#### 2.3 Performance Comparisons under different checkpoint ratio

From the above analyses, we know that the proposed scheme reduces the overhead of the system total overheads, re-computing time and the overheads of the setting checkpoints through introducing the *i*-checkpoint with low setting overheads. Next, through comparing the system total overheads with the task completion time between two-level checkpoint recovery scheme and two-level checkpoint recovery scheme under the Weibull distribution and exponential distribution, we show how the checkpoint ratio influences the system total overheads, and then show how the *i*-checkpoint influences the system total overheads. The checkpoint ratio *u = O_i_/O_m_* is the ratio of the overheads of setting *i*-checkpoint and *m*-checkpoint.

**Figure 9:**
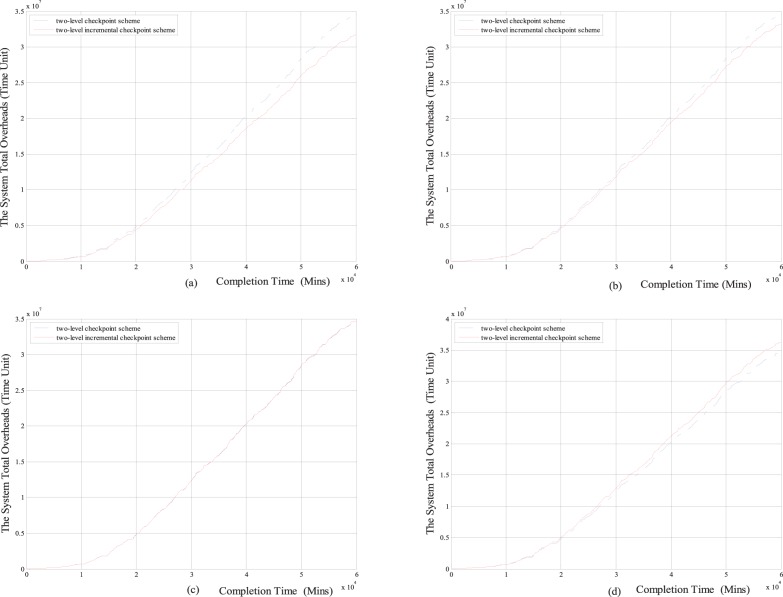
For the Weibull distribution, comparisons of the system total overheads between two-level incremental checkpoint recovery scheme and two-level checkpoint recovery scheme. (a) *u = O_i_/O_m_* = 10%; (b) *u = O_i_/O_m_* = 12.5%; (c) *u = O_i_/O_m_*  = 15%; (d) *u = O_i_/O_m_* = 17.5%.

**Figure 10:**
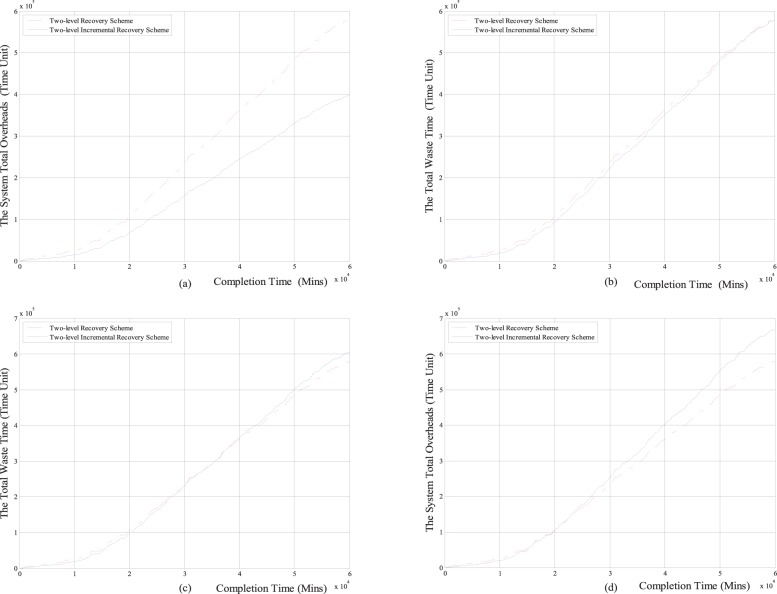
For exponential distribution, comparisons of the system total overheads between two-level incremental checkpoint recovery scheme and two-level checkpoint recovery scheme. (a) *u = O_i_/O_m_* = 10%; (b) *u = O_i_/O_m_* = 30%; (c) *u = O_i_/O_m_*  = 33%; (d) *u = O_i_/O_m_* = 40%.

From Fig. 9 and Fig. 10, we can see that when the value *u* is small, the system total overheads of two-level incremental checkpoint recovery scheme for both failure distribution types are smaller than those of the two-level checkpoint recovery scheme, for example, under the situation *u<*15% for the Weibull distribution and *u<*30% for exponential distribution. When the value *u* approaches some threshold, each checkpoint recovery scheme has its own advantages respectively, for example, when *u* approaches 15% for the Weibull distribution (the two curves coincide approximately) and 33% for exponential respectively. When the value *u* is larger than this threshold, the system total overheads of two-level incremental checkpoint recovery scheme for both failure distribution are larger than the two-level checkpoint recovery scheme, for example, when *u>*15% for the Weibull distribution and *u>*30% for exponential distribution. These conclusions are consistent with the results of the Fig. 4. This is because when the value *u* increases to a certain value, the overheads of setting *i*-checkpoints approach the overheads of setting *m*-checkpoint, which results in that the contents of *i*-checkpoint are approximately equal to the contents of *m*-checkpoint. So the *i*-checkpoint loses the advantage of low setting overhead gradually, and therefore, the advantage of the two-level incremental checkpoint becomes less and less.

In conclusion, when the value *u* is small, compared to the two-level checkpoint recovery scheme, the longer the time of long-running application is, the larger the advantage of our proposed scheme is, the larger the reduction of the system total overheads is, and the better the performance of the proposed scheme is. The introduced *i*-checkpoint in our proposed scheme only save the application states that have changed since the previous checkpoint, while the *m*-checkpoint save the total states of the application. The stored contents of the changed states are much lower than the total states, so the overhead of setting an *i*-checkpoint is much lower than the *m*-checkpoint. Therefore, the checkpoint ratio *u* can be kept in a small value. So, our two-level incremental checkpoint recovery scheme has the better performance than the two-level checkpoint recovery scheme.

## Limitations of the study, open questions, and future work

The checkpoint recovery technology has been considered as a promising technique to tolerate the system failures, guarantee the system reliability, and ensure the successful completion of the long-running tasks, and lots of checkpoint recovery schemes have been proposed recently. In this paper, based on the two-level checkpoint recovery idea, a two-level incremental checkpoint recovery scheme is proposed to further reduce the system total overheads. Three types of checkpoints, say *N*-checkpoint, *m*-checkpoint and *i*-checkpoint, are used in our scheme. The *N*-checkpoint is used to deal with the less probable or infrequent failures, while the *m*-checkpoint and *i*-checkpoint are used to deal with the more probable or frequent failures. Experiment results show that compared to the two-level checkpoint recovery scheme, the proposed scheme significantly reduces the transfers of the storing contents, the overheads of setting checkpoints and the re-computing time, and thereby reduces the system total overheads.

Unfortunately, there are still limitations in our study. Like Vaidya’s study on the two-level checkpoint recovery scheme [8], our contribution is also a theoretical idea. When Vaidya introduces his/her work, he/she just considered the ideal case and took the main performance factors into account without any practical application implemented. This does simplify the problem and pay attention to the main factors that affect the system performance basically [15], [16], [18]. Therefore, in our paper, we also adopt the same assumptions used in the works [8], [15], [16], [18] and the performance analyses focus on these main factors. This enables us to compare our scheme with the existing ones in the same circumstance, but we all know that the system performance heavily depends on the characteristics of the applications being studied. In fact, during the practical running, the checkpoint recovery scheme must be affected by other factors, such as network throughout and I/O interaction [30], [31]. Although some studies [8], [15], [16], [18] just considered the main factors and their experiments ignored the affection of those application-related factors, there have been some other researches that study their affection on the checkpoint recovery scheme [26], [27], which can be considered as another new and independent research topic. Our work focuses on the idea of the two-level incremental checkpoint recovery, and studies of the affection of these application-related factors are not our contribution of this paper.

Based on our study, four main research questions remain open and unsolved. The first is to implement our scheme in some practical application and explore how those application-related factors, such as network throughout and I/O interaction, affect the system performance. The second is to find an effective checkpoint placement method because the placement in our scheme is clearly more difficult than that in traditional one-level or two-level scheme. The third is to consider how to improve our scheme in the special case that the local storage is not error-free. The last but the most enjoyable is to introduce our idea into the multi-level checkpointing system [19] to show if a good result can be obtained. Still, our future work shall firstly focus on the implementation of our scheme in a practical system and show how the application-related factors affect the system performance.

## Conclusions

In this paper, a new two-level incremental checkpoint recovery scheme which is independent of specific failure types is proposed. By using the *i*-checkpoint with low setting overheads, compared to the two-level checkpoint recovery scheme, the proposed scheme significantly reduces the transfer of the huge memory context, the total overheads of setting checkpoints and shortens the re-computing time after the failure, and thereby reduces the system total overheads. In addition, this paper also solves the problem how to determine the optimal checkpoint placement strategy through deducing the global optimal checkpoint overheads function. The comparison results for the Weibull distribution and exponential distribution show that compared to the two-level checkpoint recovery scheme, the two-level incremental checkpoint recovery scheme proposed in this paper has the better performance, and reduces the system total overheads better. Limitations of our study are discussed, and open questions and possible future work are given.
